# Delivery of Human Adipose Stem Cells Spheroids into Lockyballs

**DOI:** 10.1371/journal.pone.0166073

**Published:** 2016-11-09

**Authors:** Karina R. Silva, Rodrigo A. Rezende, Frederico D. A. S. Pereira, Peter Gruber, Mellannie P. Stuart, Aleksandr Ovsianikov, Ken Brakke, Vladimir Kasyanov, Jorge V. L. da Silva, José M. Granjeiro, Leandra S. Baptista, Vladimir Mironov

**Affiliations:** 1 Laboratory of Tissue Bioengineering, National Institute of Metrology, Quality and Technology (Inmetro), Duque de Caxias, Rio de Janeiro, Brazil; 2 Nucleus of Multidisciplinary Research in Biology (Numpex-Bio), Federal University of Rio de Janeiro-Xerém, Duque de Caxias, Rio de Janeiro, Brazil; 3 Division of 3D Technologies, Renato Archer Center for Information Technology (CTI), Campinas, São Paulo, Brazil; 4 Institute of Materials Science and Technology, TU Wien (Technische Universität Wien), Vienna, Austria; 5 Mathematics Department, Susquehanna University, Selinsgrove, Pennsylvania, United States of America; 6 Riga Stradins University and Riga Technical University, Riga, Latvia; 7 Bioengineering Laboratory, Fluminense Federal University, Niterói, Rio de Janeiro, Brazil; University of California, San Diego, UNITED STATES

## Abstract

Adipose stem cells (ASCs) spheroids show enhanced regenerative effects compared to single cells. Also, spheroids have been recently introduced as building blocks in directed self-assembly strategy. Recent efforts aim to improve long-term cell retention and integration by the use of microencapsulation delivery systems that can rapidly integrate in the implantation site. Interlockable solid synthetic microscaffolds, so called lockyballs, were recently designed with hooks and loops to enhance cell retention and integration at the implantation site as well as to support spheroids aggregation after transplantation. Here we present an efficient methodology for human ASCs spheroids biofabrication and lockyballs cellularization using micro-molded non-adhesive agarose hydrogel. Lockyballs were produced using two-photon polymerization with an estimated mechanical strength. The Young’s modulus was calculated at level 0.1362 +/-0.009 MPa. Interlocking *in vitro* test demonstrates high level of loading induced interlockability of fabricated lockyballs. Diameter measurements and elongation coefficient calculation revealed that human ASCs spheroids biofabricated in resections of micro-molded non-adhesive hydrogel had a more regular size distribution and shape than spheroids biofabricated in hanging drops. Cellularization of lockyballs using human ASCs spheroids did not alter the level of cells viability (p › 0,999) and gene fold expression for SOX-9 and RUNX2 (*p* › 0,195). The biofabrication of ASCs spheroids into lockyballs represents an innovative strategy in regenerative medicine, which combines solid scaffold-based and directed self-assembly approaches, fostering opportunities for rapid *in situ* biofabrication of 3D building-blocks.

## Introduction

The correct delivery of mesenchymal stem cells (MSCs) to injured sites is mandatory to promote tissue repair due to their secretory capacity [1]. Adipose stem cells (ASCs) are appealing for regenerative medicine due to the relative simplicity of liposuction procedures compared to extraction techniques from the majority of other sources [2]. There is a growing consensus that the cell suspension transplant does not seem to be an appropriate way to cells delivery. Compared to single cells, MSCs and ASCs spheroids or even cell aggregates show enhanced secretion of trophic, anti-apoptotic and anti-inflammatory factors, enhancing their regenerative effects [3–6]. Recent efforts focus on the development of high-throughput methods that could improve aggregate forming efficiency, spheroid size distribution, and cell viability [7].

Spheroids are formed based on self-assembly capacity of cells through molecules recognition process. Cell spheroid assembly can be successively achieved based on spheroid fusion capacity to construct structures at tissue level. In fact, spheroids have been recently introduced as building blocks in directed self-assembly strategy [8–13]. Recent studies aimed to improve long-term cell retention and integration by using microencapsulation delivery systems with tailored biomechanical properties and that could rapidly integrate in the implantation site [14–16].

Hence, we have revisited the biofabrication process of the building blocks for bottom-up modular tissue construct, proposing the cellularization of spheroids into interlockable solid synthetic microscaffolds, so called lockyballs, recently designed and produced by two-photon polymerization. Lockyballs are spheroidal microscaffolds, small enough to be injected into tissues (200μm), specially designed with hooks and loops [17] for better retention, and multiple spheroids aggregation after transplantation. Thus, tissue constructs biofabricated from spheroids formed into lockyballs could be capable of withstanding physiological level of compression and mechanical loading after implantation.

We hypothesized that our lockyballs would provide desirable biomechanical properties whereas the interlocking mechanism would enable rapid fabrication of tissue construct *in situ* with sequential post-implantation spheroids fusion and functional tissue maturation. Recently, Danilevicius et al [18] showed an efficient cellularization of lockyballs using a mouse calvaria preosteoblastic cell line. However, the main limitation of this study relies on cell type, since cell lineages from mouse origin are not appropriate for clinical trials.

One of the main challenges of the proposed concept is the development of an effective method of biofabrication of spheroid using lockyballs, sustaining not only viability but also the differentiation potential of spheroids from cells commonly used in regenerative medicine approaches. Here we present an efficient methodology for human ASCs spheroids biofabrication into lockyballs using micro-molded non-adhesive agarose hydrogel.

## Material and Methods

### Design of lockyballs

The mechanically interlockable microscaffolds or simply “lockyballs” were designed using the graphic design software 3D STUDIO MAX (AUTODESK®) as described in our previous publication [17]. The design of lockyballs was transformed into STereoLithography STL-file suitable for additive manufacturing using open source medical image treatment software *InVesalius* which was originally developed at the Division of 3D Technology of Renato Archer Center for Information Technology (Campinas, Brazil) [http://svn.softwarepublico.gov.br/trac/invesalius].

### Fabrication of lockyballs by two-photon polymerization

Lockyball structures were produced by two-photon polymerization (2PP) of Zr-based hybrid photopolymer. The description of the material synthesis has been comprehensively reported by Ovsianikov et al. [19] and Oubaha et al. [20]. For the present experiments 0.2 wt.% of the photoinitiator (4,4'-bis(diethylamino) benzophenone (Sigma-Aldrich) was added to the material. For 2PP a Ti:sapphire laser (Femtotrain EC-800-100FS, HighQ) delivering 100 fs pulses at a repetition rate of 73 MHz at approximately 810 nm was used. The final steps were as previously reported [17]. The laser beam was focused into the material by a conventional 20 x microscope objective (NA = 0,8; Carl Zeiss). The structures were produced in a layer-by-layer fashion, with the CAD model (STL format) sliced into 1 μm thick layers. Each layer was produced by patterning in a linear scanning fashion at a distance of 0,5 μm between the neighboring scans. An average laser power of 400 mW at the scanning speed of 5 mm/s was used to induce 2PP. Approximately 2,000 lockyballs were fabricated.

### Interlockability *in vitro* tests

After the 2PP-processing, the unpolymerized material was removed by a 50/50 blend of 1-propanol and isopropanol. Isolated lockyballs were placed in large amounts (approximately 50 lockyballs) in one standard 60 mm petri dish and gently orbitally shaked at 200 rpm until they started to lock with each other (approximately 30 seconds). Three independent experiments were performed (n = 3).

### Mechanical testing (compression test)

The mechanical testing of the lockyballs (n = 10) was performed using a Microsquisher^®^ (CellScale, Canada). Detailed description of MicroSquisher^®^ is available at the company website (http://cellscale.com/products/microsquisher/). The fabricated hooks-free lockyballs were cyclically loaded at compressive force with a repetitive ramp test (5x) at 50 ⌠N, 100 ⌠N, 200 ⌠N, 400 ⌠N and 500 ⌠N and the displacement was registered using a microscope. Young’s modulus was calculated based on these measurements.

### Lipoaspirate Human Samples and Cell culture

Lipoaspirates were harvested from healthy female donors (n = 3, BMI = 25–30Kg/m^2^) who underwent abdominal liposuction for aesthetic purposes. Donor ages ranged from 18 to 45 years. Procedures received ethics approval from the Research Ethics Committee of the Clementino Fraga Filho University Hospital, Federal University of Rio de Janeiro, Brazil (Protocol 145/09). Donors provided written informed consent. Samples were stored at 4°C after surgery, and the isolation of the cells was performed within 18 hours. Adipose-derived stem cells (ASCs) were isolated and monitored by differentiation assays and flow cytometry in triplicates as previously described [21].

### Human adipose stem cells spheroids biofabrication

After expansion in monolayers, cells were harvested with trypsin and plated for spheroids biofabrication. ASCs spheroids were fabricated in hanging drops [10] or in resections of micro-molded non-adhesive hydrogel [22] using DMEM supplemented with 6.25μg/mL insulin, ITS, 1.25μg/mL bovine serum albumin (BSA), 50μg/mL ascorbic acid (all reagents from Sigma Aldrich, St. Louis, MO, USA). To generate spheroids by the hanging drop method, 2 x 10^4^ cells were suspended in 25 μL (the volume of 1 drop). A total of 30 drops (6 x 10^5^ cells) were spotted onto the underside of a lid of a 60 mm plastic culture dish. Lids were then inverted and placed onto culture dishes to create hanging drops. One spheroid was formed in each drop. For fabrication of spheroids in resections, cells were plated in micro-molded non-adhesive hydrogel (agarose 2%—UltrapureAgarose, Invitrogen, Carlsbad, CA, USA—in NaCl 0.9% solution)—molded in according to manufacturer’s recommendations (Microtissue Inc., Providence, RI, USA). Then, 2.5 x 10^5^ and 1 x 10^6^ cells were plated in micro-molded of non-adhesive hydrogel with 256 and 81 recessions, respectively. One spheroid was formed in each resection.

### Measurement of cell spheroid diameter

After 48 hours of culture, spheroids fabricated in hanging drops or in micro-molded non-adhesive hydrogel with 81 resections were photographed with a digital camera (Leica DFC 500) coupled to an optical inverted microscope (Leica DMI 6000B). We randomly measured a total of 45 spheroids from 2 or 3 micro-molded non-adhesive hydrogel. For the hanging-drop assay, we randomly measured a total of 45 spheroids from 2 or 3 petri-dishes. Both assays were performed in triplicate from three different cell samples (n = 3). Major (D) and minor (d) spheroids diameter were determined using Image J software. The shape elongation coefficient (*k*) of the fabricated spheroids was calculated by the ratio D/d.

### Human adipose stem cells spheroids biofabrication into lockyballs

A total of 180 lockyballs were plated in the micro-molded hydrogel with 256 resections. Subsequently, 2.5 x 10^5^ cells were seeded in micro-molded hydrogel in order to allow spheroids formation into lockyballs. The assay was performed in triplicate from three different cell samples (n = 3). All subsequent analyses were performed after 2–3 days. DAPI staining and the intrinsic fluorescence of lockyballs were visualized by confocal fluorescence microscopy using a band-pass filter for the detection of both dyes and by fluorescence microscopy followed by deconvolution.

### Estimation of spheroids viability

Spheroids formed in micro-molded hydrogel in the absence and into lockyballs were collected (n = 90) after a careful selection of resections carrying only 1 lockyball. Spheroids were stained by DAPI for nuclei integrity evaluation. For quantitative assay, cell spheroids were dissociated using triple express solution (Invitrogen). Cells suspension was incubated with 7AAD-staining (BD Biosciences, Franklin Lakes, NJ, USA) and analyzed by flow cytometry (BD Accuri™ C6). No gate strategy was performed on FCS versus SSC distribution, including all events for 7AAD positivity cell analysis (gated in R1). Viable cells were identified by 7AAD positive cell exclusion (events outside R1) and 20,000 events were acquired in each tube. Three independent experiments were performed (n = 3).

### Microscopic analysis

An inverted optical microscope (AxioObserver Z1, Zeiss) was used for light and confocal images (LSM700, Zeiss). All 3D confocal images were generated with specialized microscopy software (Zen, Zeiss). Other fluorescence images were acquired using a fluorescence microscopy followed by deconvolution (Leica DMI 6000). For the scanning electron microscopy (Quanta FEI), the samples were sputtered with a 15 nm thick Pd/Au coating.

### Quantitative real-time PCR (qPCR)

Spheroids formed in micro-molded hydrogel in the absence and into lockyballs were collected (n = 90) after a careful selection of resections carrying only 1 lockyball. Spheroids were incubated with RLT buffer (Qiagen, Sweden). RNA extraction was performed with RNeasy Mini Kit according to manufacturer’s instructions(Qiagen, Sweden). Gene expression levels involved in regulation chondrogenic, SRY (sex determining region Y)-box 9 (SOX9), and osteogenic, Runt-related transcription factor 2 (Runx2), were evaluated. Total RNA from each condition was extracted with the QIAGEN RNeasy® mini kit. The qPCR was carried out using the AgPath-ID™ one-step RT-PCR kit. In brief, 1,5 μl total RNA (15ng/μl) was reverse transcribed and amplified in master mix composed by 5 μl of 2x RT-PCR buffer, 0.4 μl of 25x RT-PCR enzyme mix, 0,67 μl of Detection Enhancer and completed with RNase free water to a final volume of 10 μl reaction mixture. Specific primers and TaqMan® probe were performed from Assay-on-Demand Gene Expression Products (Applied Biosystems). Each RNA sample of the gene was run in triplicate and normalized to the expression of the housekeeping gene RPL using the ΔΔCt method. In order to compare differences fold expression, relative expression levels were calculated for each sample after normalization. The assays were performed in triplicate (n = 3) from two different cell samples (n = 2).

### Statistical analysis

A non-parametric Student´s t test was used to compare the percentage of viable cells in spheroids formed in the absence and into lockyballs. The results presented are expressed as the mean ± standard error of the mean, and *p* <0.05 was considered statistically significant. A parametric Student´s t test was used to compare gene fold expression in spheroids formed in the absence and into lockyballs. The analyses were performed using the software GraphPad Prism 6.0 (GraphPad Software, San Diego, CA, USA).

## Results

### Fabrication, material properties and the *in vitro* behavior of lockyballs

**Fig 1:**
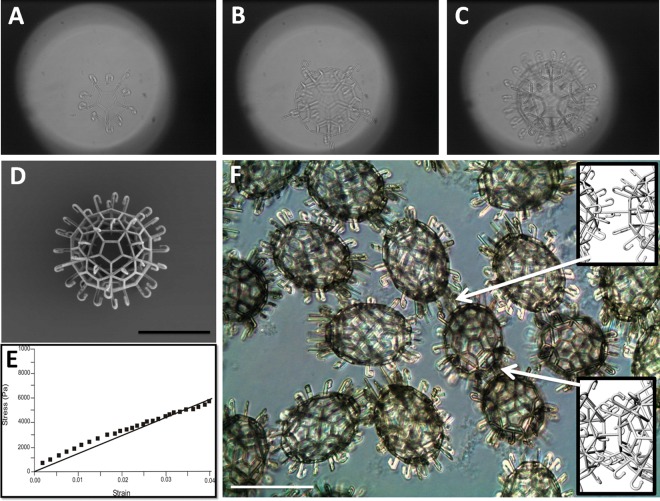
Fabrication, material properties and the *in vitro* behavior of lockyballs. (A, B, C) Sequential steps of layer-by-layer fabrication of lockyball using two-photon polymerization from photosensitive biomaterial. (D) Scanning electron microscopy image of a fabricated lockyball. (E) Representative results of estimation of lockyball stress-strain relationship using compression test performed with MicroSquisher^®^ (CellScale, Canada). The stress-strain relationship is typical of solid polymers. (F) Interlocked lockyballs demonstrating two types of interlocking mechanisms: hook-to-hook and hook-to-loop (arrows). Phase contrast. Details of both types of interlocking mechanisms are illustrated in inserts on the right side of image. Bars size: 100 micrometers.

By using 2PP, lockyballs could be produced directly from the CAD input. The 3D model of a lockyball in a STL format was sliced into layers in order to generate the according set of coordinates defining the scanning trajectory for the laser beam, i.e., the polymerized regions. Due to slight material shrinkage [23], a reduction of lockyball diameter of about 4% was observed, when compared to CAD model dimensions. Sequential steps of lockyballs fabrication process are presented in Fig 1A–1C.

Lockyballs are empty (hollow) microscaffolds with porous wall with interlockable hooks (Fig 1D). The mechanical strength of fabricated lockyballs was estimated. The representative stress-strain relationship of a single measurement demonstrates a linear relationship, typical of solid polymers (Fig 1E). The Young’s modulus was calculated at level 0.1362 +/- 0.009 MPa. The interlocking *in vitro* test demonstrates high level of loading induced interlockability of fabricated lockyballs. Both the relatively flexible and lose hook-hook, and the more rigid and tight hook-loop (using elevated pentagons) interlocking mechanisms were observed (Fig 1F).

### Biofabrication of human ASCs spheroids

**Fig 2:**
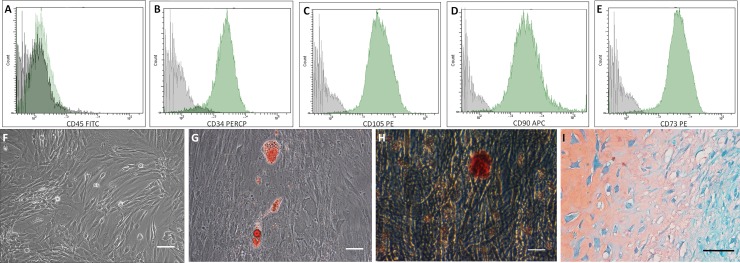
Adipose tissue derived stem cells (ASCs) characterization. (A–E) ASCs used in this study showed the typical surface marker profile CD45 negative, CD34, CD105, CD90 and CD73 positives (Green histograms). Gray histogram—negative controls. (F) Monolayer of culture-expanded ASCs. Phase contrast. (G–I) ASCs are multipotent for adipogenic, osteogenic and chondrogenic lineages. (G) Oil Red O staining was used to reveal intracellular lipid droplets, phase contrast. (H) Alizarin Red staining was used to reveal extracellular calcium depots, phase contrast. (I) Safranin staining was used to reveal sulfated glycosaminoglycan content from pellets cultures. Optical microscopy. Bar size: 100 micrometers.

ASCs expressed surface markers (Fig 2A–2E) and showed a fibroblastic morphology (Fig 2F) typical of *in vitro* mesenchymal cells. They were able to differentiate towards the adipogenic, osteogenic and chondrogenic lineages as evidenced by Oil Red O, Alizarin Red and Safranin stains, respectively (Fig 2G–2I).

**Fig 3:**
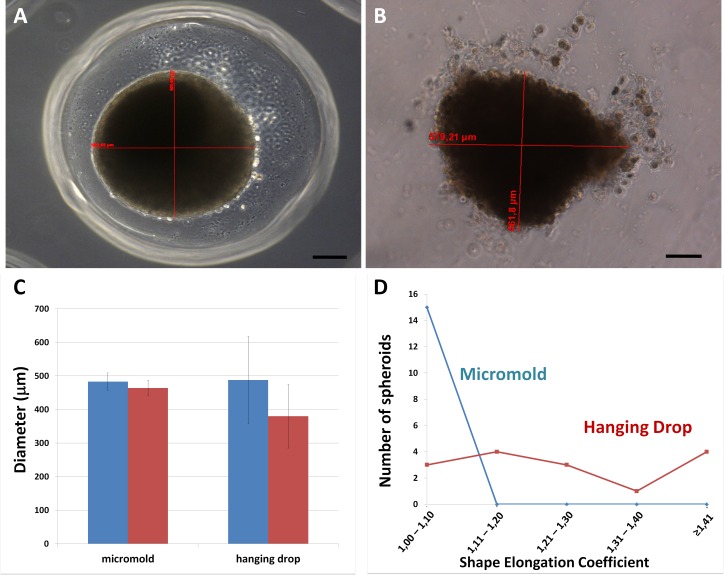
Biofabrication of human ASCs spheroids. (A) ASCs spheroid fabricated in a confined space (resection) of micro-molded non-adhesive hydrogel has more regular shape and size than (B) ASCs spheroid fabricated by the hanging drop method. Phase contrast. Bar size: 100 micrometers. (C) Graph showing major (blue bar) and minor (red bar) diameters of spheroids generated in hanging drop and in resections of micro-molded non-adhesive hydrogel. Note that standard deviation in hanging drops is higher. A total of 45 spheroids were measured randomly. Graph represents the mean ± standard error (D) Graph showing shape elongation coefficient (major/minor diameter of each spheroid) distribution of spheroids biofabricated by the two techniques representative from 1 micro-molded non-adhesive hydrogel and 1 petri-dish. (blue: spheroids fabricated in resections of micro-molded hydrogel; red: spheroids fabricated in hanging drops).

Human ASCs plated in hanging drops or in resections of micro-molded non-adhesive hydrogel formed spheroidal aggregates. Phase contrast images of spheroids fabricated using both methods revealed pertinent differences (Fig 3A and 3B). Spheroids in resections show a more defined border, while the ones fabricated in hanging drops had some peripheral cells detached from the central structure. Each spheroid presented a major and a minor diameter (Fig 3C), which was measured for shape elongation coefficient determination. Diameters´ values were more variable in hanging drop spheroids, observed by the increased standard deviation of the mean compared to spheroids generated in hydrogel resections (Fig 3C). Micro-molded non-adhesive hydrogel provides spheroids with a coefficient around 1,0–1,1, that reflects a more regular shape relative to the hanging drop method (Fig 3D).

### Biofabrication of human ASCs spheroids into lockyballs

**Fig 4:**
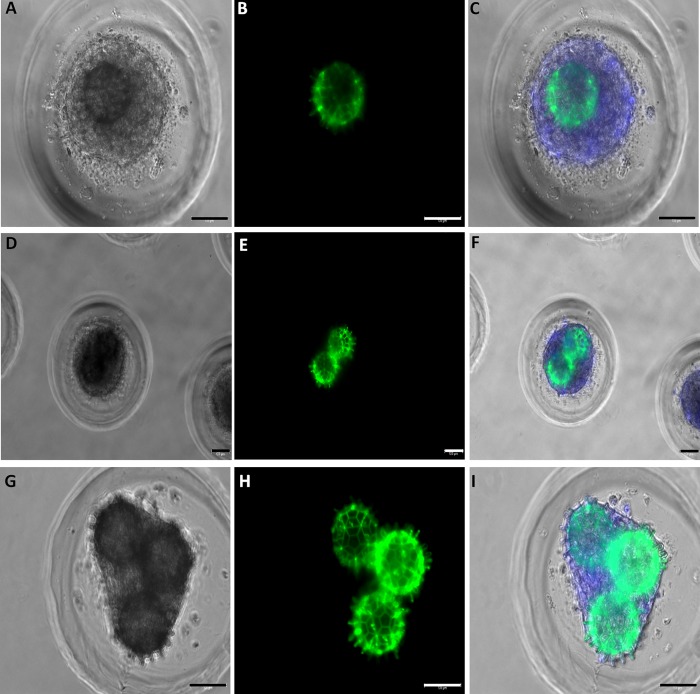
Biofabrication of human ASCs spheroid into lockyballs. Micro-molded resections showing (A, B, C) one, (D, E, F) two or (G, H, I) three lockyballs. Note that all spheroids are inside lockyballs. (D-I) Resections showing interlocking. (A, D, G) Light microscopy, (B, E, H) Green: autofluorescent lockyballs due to autofluorescence of photo-polymerized biomaterial (C, F, I) Merge of pictures: light microscopy, DAPI staining (blue), green. Bar size: 100 micrometers.

The mathematical modeling and computer simulation using *Surface Evolver* software indicate a 25% of compaction during spheroid formation after cell plating [17]. Therefore, lockyballs must be completely covered with cells during cellularization and, after cell compaction spheroids could be formed inside lockyballs. Indeed, human ASCs spheroids were formed into lockyballs by seeding cell suspension in micro-molded non-adhesive agarose hydrogel. Lockyballs cellularization did not jeopardize their interlocking (Fig 4D–4I), although the cells covered lockyballs´ boarder hooks (Fig 4A–4I). In addition, it was possible to note that the resulting spheroid diameter is closer to the diameter of two interlocked lockyballs (Fig 4D–4F)

**Fig 5:**
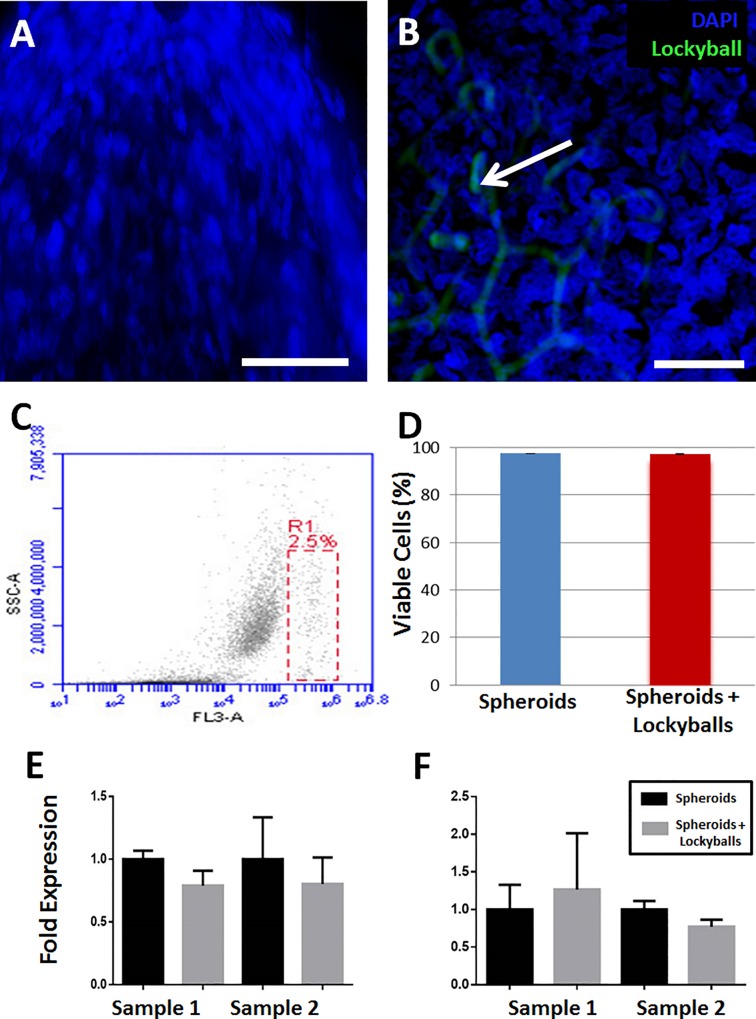
Viability of human ASCs spheroids formed into lockyballs. (A, B) DAPI staining of human ASCs spheroid formed in the absence (A) and into (B) lockyballs (blue: DAPI staining, green: autofluorescent lockyballs) shows cell nuclei without any detectable morphological signs of apoptosis or cell death. Bar size: 50 micrometers. (C) Viable cells identified by 7AAD exclusion (dead cells in R1) using flow cytometry for quantitative analysis. Dot-plot graph is representative of digested mass from 90 spheroids in the absence and into lockyballs. Twenty thousand events were acquired in each tube. (D) Percentage of viable cells in spheroids in the absence (blue bar; 97,6±1,1) and into (red bar; 97,3±1,2) lockyballs. Graph represents the mean ± standard error of the mean of three independent experiments (*p* › 0,99). Fold expression for SOX9 (E) and for RUNX2 (F) master genes in spheroids in the absence and into lockyballs. (E, F) Graphs represent the mean ± standard error of the triplicates from two independent cell samples. *Students´ t* test was used and resulted a *p* = 0,1965 (sample 1) and *p* = 0,6444 (sample 2) for SOX9; *p* = 0,7620 (sample 1) and *p* = 0,1953 (sample 2) for RUNX2.

DAPI staining revealed nuclei integrity of cells in spheroids formed in the absence and into lockyballs (Fig 5A and 5B). We have performed flow cytometry analysis as a complementary assay to DAPI staining. Flow cytometry analysis of digested mass from spheroids showed that lockyballs did not significantly alter their percentage of viability (Fig 5C and 5D). More importantly, spheroids formed into lockyballs did not alter significantly the fold expression for the genes SOX9 and RUNX2 (Fig 5E and 5F) known as master genes for chondrogenesis and osteogenesis, respectively.

## Discussion

The main outcome of the present study was the development of an efficient biofabrication method of spheroids from human ASCs into interlockable microscaffolds or lockyballs, to serve as building blocks for bottom-up modular directed tissue self-assembly. The biofabrication of spheroids into lockyballs does not compromise cell viability and master genes expression for chondrogenic and osteogenic lineages while facilitating their capacity of *in vitro* aggregation among multiple spheroids.

There are different fabrication technologies of microscaffolds and microcarriers for regenerative medicine approaches [24–30]. However, the method presented in this study using 2PP technology is regarded as one of the most advanced methods due to its high resolution and unique capacity to make a desirable and precise design of microscaffolds using CAD softwares. 2PP is able to fabricate parts with a resolution of 100 nm or more [31,32]. In the case of lockyballs, the 2PP potential resolution level is suitable for the large scale production of the 3D structures named as 'lockyballs', with an approximate diameter of 200 μm.

The high spatial resolution of 2PP allows the fabrication of hooks, which are important functional features that provide the locking ability of the microscaffolds. Hook-loop interlocking provides more rigid fixation whereas hook-hook mechanism facilitates some flexibility and mobility of interlocked microscaffolds. As previously discussed, interlocking ability does not require co-positioning or co-alignment of lockyballs, enabling effective interlocking of randomly placed microscaffolds in 3D space.

The estimation of mechanical properties of microscaffold is not a trivial research task due to their meso-structural scale. The data presented here demonstrated that the mechanical property of lockyballs is higher than the biomechanical property of spheroids measured by the same equipment reported in another study [33]. We could postulate that lockyballs will provide tissue construct with tunable and desirable enhanced biomechanical properties. In order to reach full tissue regeneration *in vivo*, the scaffold should be degradable. Heretofore lockyballs design did not allow the use of a diversity of materials, resulting in a non-degradable microscaffold.

The interlocking capacity of lockyballs is an inherent property of their design with hooks and loops. The influence of lockyballs number, speed and time of shaking were not evaluated in this study. However, the interlocking capacity was also observed during cellularization assays. Resections of micro-molded non-adhesive hydrogel showing two or more lockyballs revealed an interlocking capacity in the absence of shaking.

The undesirable problems using hanging drops observed in this study include the simultaneous formation of several aggregates around a central spheroid in one hanging drop and the irregular distribution of both spheroid size and shape. On the other hand, the space confinement provided by the hydrogel resection diameter forces the formation of a single spheroid, with a more regular distribution of spheroid size and shape, besides favoring spheroid formation into lockyballs. Due to our manual handling, it is not possible to control the number of lockyballs per resection. This limitation could be overcome using automation for cells and lockyballs seeding.

Furthermore, micro-molded technology brings opportunities to a sort of applications including drugs and nanoparticle testing, cell therapy, tissue engineering and biofabrication [34–36], mainly because of the possibility of hundreds of spheroids biofabrication in a unique micro-molded with homogeneous size and shape. Using micro-molded non-adhesive hydrogel also opens the possibility of cell seeding automation, since cell suspension is dispensed in an isolated well of cell culture plate. The automation of cell seeding will allow the standardization of a scalable spheroids biofabrication.

**Fig 6:**
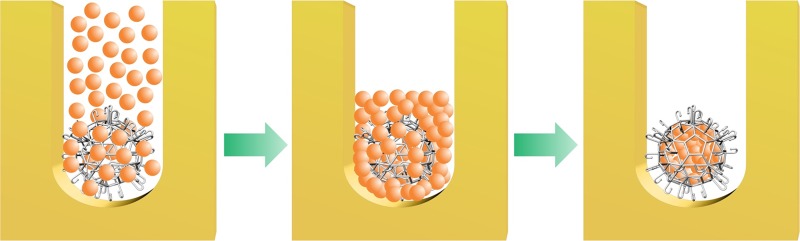
Representative scheme of cell seeding into lockyball using micro-molded non-adhesive hydrogel. Scheme demonstrating cell seeding of lockyballs placed into resections. As a direct result of tissue compaction in confined space (resections), spheroids formed into lockyballs could be reproducibly biofabricated with maximal cell density inside lockyballs.

Porosity of lockyball walls allowed efficient cellularization in micro-molded confined space occupied by spheroids. This approach guaranteed high-density cell seeding inside lockyballs. However, effective spheroid formation into lockyballs using micro-molded non-adhesive hydrogel method needs careful consideration including: i) the ratio between the diameter of resection and the external diameter of microscaffold, ii) the ratio between microscaffold pore diameter and cell diameter, and, finally, iii) theoretically calculated 25% retraction of cell aggregates during their transition into a more cohesive spheroid [17] (Fig 6).

Besides, spheroids formed into lockyballs showed comparable levels of nuclei integrity and viability also maintaining fold gene expression for chondrogenesis and osteogenesis master genes. We and another study [4] performed viability assay after spheroids digestion in order to allow their analysis by flow cytometry. The main limitation relies on a few dead cells being lost during digestion and centrifugation steps. However, our focus was to compare both experimental conditions (spheroids in the absence or into lockyballs). Our previous study [18] also reported an efficient cellularization with a considerable cell viability using a pre-osteoblastic lineage into lockyballs. In this study, beyond cellularization and viability parameters, we showed maintenance in ASCs differentiation potential for chondrogenic and osteogenic lineages by gene expression analysis. Although not being a conclusive result of a complete differentiation into specialized cells, the gene expression comprises the cell potential to important differentiation pathways for tissue engineering. Osteogenic and chondrogenic assays of ASCs spheroids into lockyballs for *in vivo* experiments are in course.

Finally, the proposed tissue building-blocks biofabrication, based on spheroids formed into lockyballs could provide several important advantages, such as: i) it allows rapid interlocking of spheroids; ii) it could provide fabricated tissue construct with tunable and desirable enhanced mechanical properties; iii) it opens opportunities for rapid *in situ* tissue construct biofabrication; and, iv) it could potentially enhance the integration of spheroids with surrounding tissue at the implantation site.

## Conclusions

The biofabrication of ASCs spheroids into lockyballs represents an innovative strategy in regenerative medicine, by combining solid scaffold-based and directed tissue self-assembly approaches, for an efficient spheroids delivery and engraftment. Besides, it enables rapid *in situ* biofabrication of 3D building-blocks.

## Abbreviations

MSCs: Mesenchymal stem cells
ASCs: Adipose stem cells
2PP: two-photon polymerization
DAPI: 4',6-diamino-2-phenylindole
7AAD: 7-aminoactinomycin D
STL: Stereolithography
CAD: computer aided design
ITS: Insulin-Transferrin-Selenium
DMEM: Dulbecco´s Modified Eagle´s Medium
BSA: Bovine Serum Albumin
